# Multiprofessional perinatal care in a pregnant patient with acute respiratory distress syndrome due to COVID-19

**DOI:** 10.1186/s12884-021-04059-y

**Published:** 2021-08-26

**Authors:** Pilar Palmrich, Bernhard Roessler, Lukas Wisgrill, Stephanie Kampf, Pia Gattinger, Rudolf Valenta, Edith Fleischmann, Angelika Berger, Herbert Kiss, Alex Farr

**Affiliations:** 1 Department of Obstetrics and Gynecology, Division of Obstetrics and Feto-Maternal Medicine, Comprehensive Center for Pediatrics, Medical University of Vienna, Waehringer Guertel 18–20, A-1090 Vienna, Austria; 2 Medical Simulation and Emergency Management Research Group, Department of Anesthesia, Intensive Care Medicine and Pain Medicine, Medical University of Vienna, Vienna, Austria; 3 Department of Pediatrics and Adolescent Medicine, Division of Neonatology, Intensive Care Medicine and Neuropediatrics, Comprehensive Center for Pediatrics, Medical University of Vienna, Vienna, Austria; 4 Department of Surgery, Division of General Surgery, Medical University of Vienna, Vienna, Austria; 5 Department of Pathophysiology and Allergy Research, Division of Immunopathology, Center for Pathophysiology, Infectiology and Immunology, Medical University of Vienna, Vienna, Austria; 6 NRC Institute of Immunology FMBA of Russia, Moscow, Russia; 7 Laboratory for Immunopathology, Department of Clinical Immunology and Allergy, Sechenov First Moscow State Medical University, Moscow, Russia; 8 Karl Landsteiner University of Health Sciences, Krems, Austria

**Keywords:** ARDS, COVID-19, Perinatal care, Pregnancy, SARS-CoV-2, Transmission

## Abstract

**Background:**

The coronavirus disease (COVID-19) pandemic has caused ongoing challenges in health services worldwide. Despite the growing body of literature on COVID-19, reports on perinatal care in COVID-19 cases are limited.

**Case presentation:**

We describe a case of severe acute respiratory distress syndrome (ARDS) in a 36-year-old G5/P2 pregnant woman with morbid obesity, confirmed severe acute respiratory syndrome coronavirus 2 infection, and fulminant respiratory failure. At 28^+ 1^ gestational weeks, the patient delivered an uninfected newborn. Using ImmunoCAP ISAC® technology, we found no immunoglobulin (Ig) M antibodies, suggesting that no mother-to-child viral transmission occurred during pregnancy or delivery. The maternal respiratory state improved rapidly after delivery; both maternal and neonatal outcomes were encouraging given the early gestational age and fulminant course of respiratory failure in our patient.

**Conclusions:**

The management of ARDS in pregnant women with COVID-19 is complex and requires an individualized, multidisciplinary approach, while considering maternal and fetal outcomes.

## Background

The coronavirus disease (COVID-19), caused by the severe acute respiratory syndrome coronavirus 2 (SARS-CoV-2), is a global health emergency. Despite the increasing number of publications about COVID-19, reports on the management of pregnant women with the disease are still limited. This is crucial because pregnancy is known to be a state of partial immunosuppression, causing a relative susceptibility to viral infections [1]. Data from prior epidemic respiratory illnesses, such as those caused by the influenza A virus H1N1, have demonstrated an increased risk of severe morbidity and mortality during pregnancy. However, there is no consensus on the management of pregnant women with COVID-19 [2–4]. Herein, we report a case of a pregnant woman at 27^+ 5^ gestational weeks, who presented with fulminant acute respiratory distress syndrome (ARDS) due to severe COVID-19.

## Case presentation

### Primary care

In October 2020, a 36-year-old woman presented at 27^+ 5^ gestational weeks at a local hospital with new-onset symptoms of fever, cough, mild dyspnea, and ageusia aside from her class III obesity (body mass index = 50.7 kg/m^2^), she had no other comorbidities or a pertinent medical history. The patient, G5/P2, had an obstetric history of two uncomplicated term cesarean sections and two early miscarriages. The course of her ongoing pregnancy had been unremarkable at that time. The woman reported normal fetal movements and no symptoms such as uterine contractions, amniotic fluid leakage, or vaginal bleeding. A real-time polymerase chain reaction (RT-PCR) test using nasopharyngeal swabs was positive for SARS-CoV-2 and negative for influenza A, B, and respiratory syncytial virus (RSV). She was discharged from the hospital for self-quarantine because of her stable respiratory condition.

**Fig. 1:**
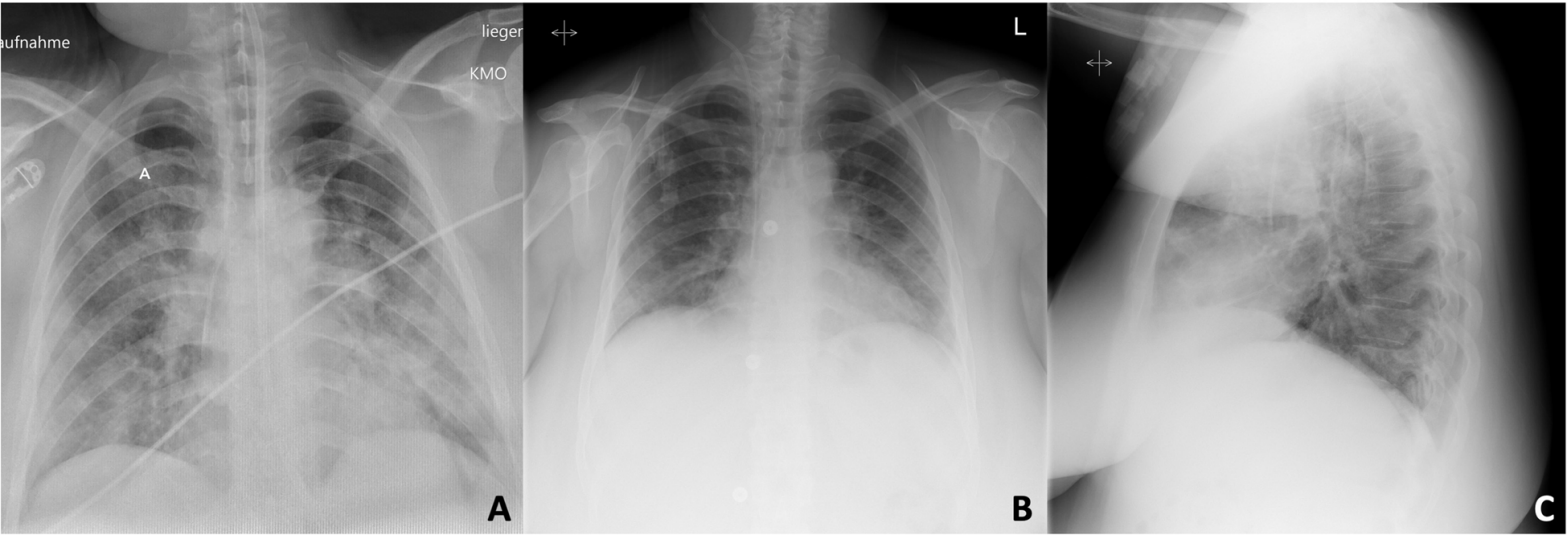
Chest radiography in a patient with ARDS due to COVID-19 before and after cesarean section: **A** on postoperative day 1, showing bilateral confluent opacities with basal accentuation; **B** on postoperative day 10, showing decreasing bilateral consolidations (ARDS: acute respiratory distress syndrome; COVID-19: coronavirus disease)

**Fig. 2:**
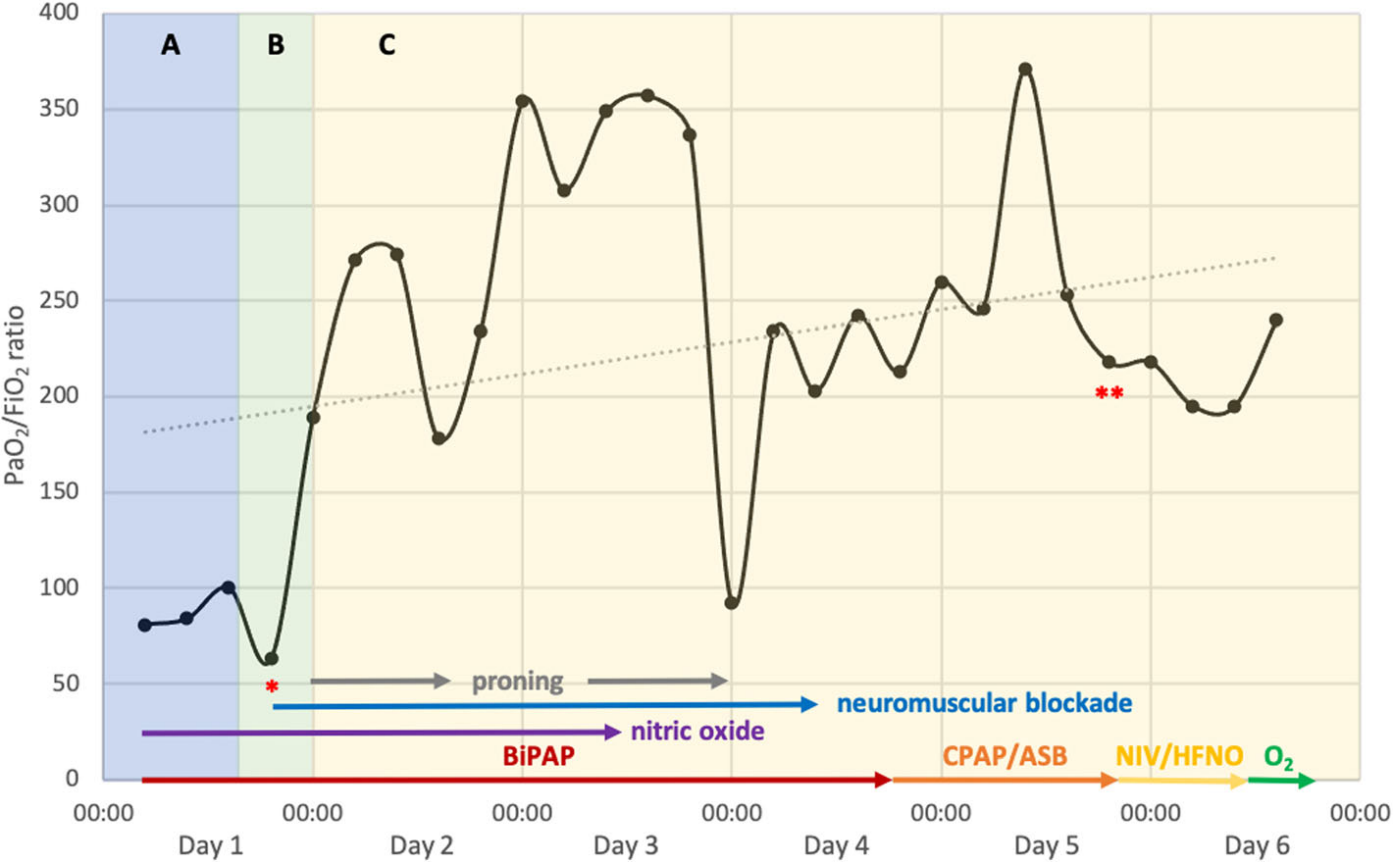
PaO_2_/FiO_2_ (P/F) ratio during ventilation in a patient with ARDS due to COVID-19 before (**A**), during (**B**) and after (**C**) cesarean section. The (*) marks the time point of delivery, and (**) marks the time point of extubation (ARDS: acute respiratory distress syndrome; BiPAP, biphasic positive airway pressure; CPAP/ASB, continuous positive airway pressure/assisted spontaneous breathing; NIV/HFNO, non-invasive ventilation/high-flow nasal oxygen therapy)

However, she presented with worsening symptoms of respiratory distress the next day, with the following vital signs: respiratory rate: 50/min; SpO_2_: 89% on room air; blood pressure: 87/49 mmHg; and heart rate: 115 bpm. She was admitted to the intensive care unit (ICU) where non-invasive ventilation therapy was initiated. She received intravenous fluids and a low dosage of supportive catecholamines, and was started on ampicillin/sulbactam for antibiotic treatment of suspected bacterial coinfection. Weight-adapted prophylactic doses of low-molecular-weight heparin (LMWH) and enoxaparin (40 mg twice daily) were administered. Chest radiography showed bilateral opacities in the lower lobes and left middle lobe, consistent with pneumonia (Fig. 1 A–C). Laboratory test results showed lymphopenia, eosinophilia, anemia, and elevated C-reactive protein and lactate dehydrogenase levels (Table 1). Maternal echocardiography revealed no signs of cardiac failure. Fetal sonography and cardiotocographic monitoring showed no signs of fetal distress. Antenatal corticosteroids were administered to accelerate fetal lung maturation due to the risk of preterm birth. On day 2 of hospitalization and at 28^+ 0^ gestational weeks, her respiratory condition worsened; she was consequently intubated and transferred to a larger hospital. Despite maximum ventilatory support, the patient developed further deterioration of oxygenation and decarboxylation with a PaO_2_/FiO_2_ (P/F) ratio of 84; therefore, extracorporeal membrane oxygenation (ECMO) therapy was considered a possible rescue therapy. On day 3 of hospitalization, she was transferred to our tertiary center for ECMO evaluation. The patient initially presented with a PF ratio of 84, receiving biphasic positive airway pressure (BiPAP) and requiring a FiO_2_ of 1.0, peak pressure of 35 mbar, and positive end-expiratory pressure (PEEP) of 18 mbar, resulting in a tidal volume of 334 mL. However, local ECMO entry criteria based on the EOLIA Trial by Combes et al. (PF ratio < 80 for > 6 h among others) were not met upon admission; thus, the patient was not started on ECMO treatment [5]. Instead, rescue therapy with nitric oxide was initiated, which improved the PF ratio (Fig. 2). Fetal sonography performed upon arrival showed an estimated fetal weight of 1578 g, and normal umbilical artery Doppler values, and amniotic fluid measurements.

**Table 1:** Laboratory values during intensive care of a COVID-19 patient with ARDS pre−/post-cesarean section

Measure	Reference Range	Day 1	Day 2	Day 3	Day 4 POD 1	Day 5 POD 2	Day 6 POD 3	Day 7 POD 4	Day 8 POD5	Day 9 POD 6
White blood cell count *(G/L)*	4.0–10.0	9.52	16.4	11.4	10.7	10.1	9.22	9.75	9.73	10.44
Lymphocyte count *G/L*	1.1–4.0	0.98	0.97	0.68	1.61	1.92	1.01	1.65	1.65	2.19
Interleukin-6 (pg/mL)	≤ 7	n.a.	37.8	22.2	263	47.2	23.7	30.4	24	12.7
C-reactive protein *(mg/dL)*	< 0.5	11.5	12.9	7.57	6.28	11.61	9.73	7.2	7.89	6.03
Procalcitonin (ng/mL)	< 0.5	n.a.	0.2	0.1	0.46	6.68	4.17	2.28	1.24	0.69
LDH (U/L)	< 250	n.a.	n.a.	332	333	321	267	354	323	309
D-dimer (μg/ml)	< 0.5	n.a.	1.1	1.86	1.67	0.69	0.74	0.86	0.97	1.45

*LDH* lactate dehydrogenase, *POD* Postoperative day, *n.a*. Not available

### Perinatal care

Given the patient’s unstable condition, risk for further respiratory and hemodynamic decompensation, and promising fetal condition, a multidisciplinary expert consultation with anesthesiologists, obstetricians, and neonatologists agreed to proceed with a cesarean section. The patient was started on magnesium sulfate for fetal neuroprotection. On day 4 of hospitalization, at 28^+ 1^ gestational weeks, the cesarean section was conducted in the ICU in compliance with strict safety guidelines, using full personal protective equipment. A liveborn male neonate weighing 1445 g was delivered with an Apgar score of 3/7/7 at 1, 5, and 10 min. The amniotic fluid was clear, while intraoperative blood loss was estimated to be 500 mL. The patient received oxytocin intraoperatively and postpartum to prevent uterine atony, as well as a single-shot antibiotic prophylaxis with cefazolin.

### Intensive care

Following the delivery, the patient’s respiratory state improved within a few hours. She required two cycles of prone positioning, as well as continuous neuromuscular blockade, which led to a significant improvement in the P/F ratio (Fig. 2). The patient was started on a 5-day-treatment with remdesivir and dexamethasone according to our local protocol. Dostinex was administered for primary weaning. A weight-adjusted prophylactic dose of LMWH was re-initiated 6 h after delivery. On postoperative day (POD)-3, a continuous positive airway pressure (CPAP) was initiated. Uncomplicated weaning allowed extubation on POD-5. Supportive catecholamine therapy was discontinued simultaneously. The patient remained normotensive and hemodynamically stable, and received intermittent CPAP mask therapy.

Due to fever peaks, antibiotics were changed to amoxicillin/clavulanic acid on POD-2 and discontinued on POD-5, after bacterial cultures of blood, urine, and bronchial lavage showed no abnormal findings. Due to dermatomycosis of the thighs and the lower abdomen at the incision site, the patient received oral fluconazole. On POD-7, 11 days after onset, she was deisolated after three RT-PCR tests showed a cycle threshold value above 30, with the final nasopharyngeal swab being negative for SARS-CoV-2. On the same day, she was transferred to the maternity ward with low-flow oxygen insufflation via a nasal cannula (1 L/min, SpO_2_ 98%). The patient was discharged in good clinical condition on POD-13.

### Neonatal care

The newborn was intubated immediately after delivery and transferred to the neonatal ICU due to respiratory distress and insufficient breathing effort. Surfactant (200 mg/kg) was administered intratracheally with immediate improvement in respiratory condition. The next day, she was extubated and received respiratory support via CPAP, followed by a nasal high-flow cannula. Repeated nasal swabs and tracheal aspirates over the first 5 days of life were all negative for SARS-CoV-2. The clinical and laboratory signs of perinatal infections were also negative. Prophylactic antibiotics with ampicillin/gentamicin were discontinued after 48 h. Oral feeding was well tolerated, while postnatal growth was based on the corresponding percentiles. At the corresponding age of 31^+ 1^ weeks, the infant was transferred to a hospital closer to the parents’ residence in a clinically stable condition with low respiratory support (0.21% FiO_2_: 4 L/min), receiving full enteral feeding. The newborn was finally discharged from the hospital at the age of 34^+ 5^ weeks.

### Placental transmission

**Fig. 3:**
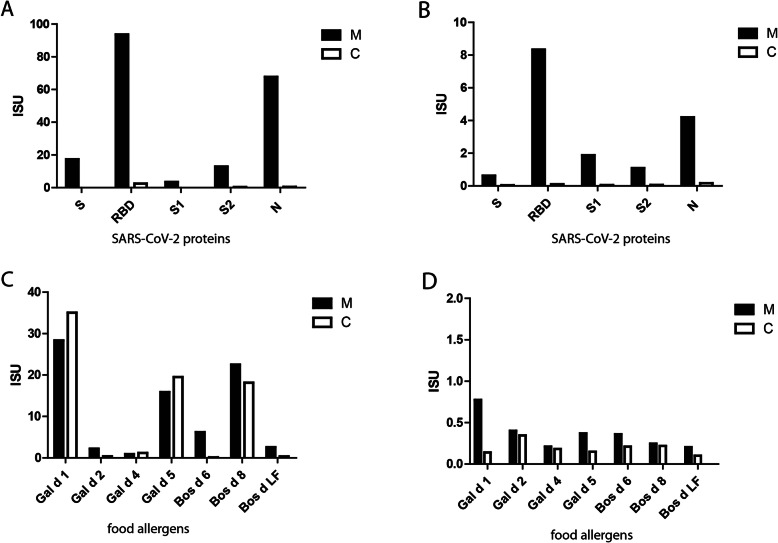
SARS-CoV-2 specific immunoglobulin reactivity in a patient with ARDS due to COVID-19: **A** IgG and **B** IgM reactivity to SARS-CoV-2 proteins in the mother (black bars) and child (white bars); IgG **C** and IgM **D** reactivity to food allergens in the mother (black bars) and child (white bars). Y-axis: ImmunoCAP ISAC® standardized units (ISU) correspond to bound immunoglobulin. Measurements were performed in triplicates; mean values are shown (COVID-19: coronavirus disease; SARS-CoV-2: severe acute respiratory syndrome coronavirus 2; Ig: immunoglobulin)

SARS-CoV-2-specific immunoglobulin reactivity and transplacental transmission were investigated by determining IgG and IgM to the SARS-CoV-2 spike protein (S), receptor binding domain (RBD), nucleocapsid protein (N), and spike protein subunits (S1, S2), as well as to a panel of egg-and milk-derived food allergens (*Gal-d-1*, *Gal-d-2*, *Gal-d-4*, *Gal-d-5*, *Bos-d-6*, *Bos-d-8*, and *Bos-d-LF*) using ImmunoCAP ISAC® [6]. The ability of maternal antibodies to inhibit the binding of RBD to angiotensin-converting enzyme 2 (ACE2) was studied using a recently developed molecular interaction assay [7]. On POD-9, antibody testing showed a strong IgG and IgM reactivity to S, RBD, and N in the maternal, but not in the neonatal, serum sample (Fig. 3A–B). When the maternal serum was tested for the presence of antibodies that could inhibit the binding of RBD to ACE2, there was a strong inhibition of > 70%. The lack of SARS-CoV-2 specific IgM in infants and negative RT-PCR test results suggest that there was no vertical transmission during pregnancy or delivery. Despite the absence of SARS-CoV-2 specific IgG in the neonatal serum sample, high levels of IgG to food allergens *Gal-d-1*, *Gal-d-5,* and *Bos-d-8* were detected (Fig. 3B), indicating the transfer of maternal IgG across the placenta.

## Discussion and conclusions

Although the currently available data mostly suggest mild courses of the disease during pregnancy, the management of ARDS is particularly challenging in these patients. Our patient met the criteria for severe ARDS due to COVID-19 with refractory hypoxemia, despite optimized mechanical ventilation in evaluation for ECMO therapy. Considering the decompensating maternal condition and early gestational age, both maternal and fetal outcomes needed to be considered. The current management of ARDS consists of supportive care, including low tidal volume ventilation, neuromuscular blockade, and prone positioning in moderate-to-severe ARDS [8]. In cases of worsening, refractory hypoxemia (P/F ratio < 80 for > 6 h, or < 50 for > 3 h), patients should be considered for ECMO [9]. Pregnant women have an increased risk of developing ARDS requiring mechanical ventilation compared to non-pregnant women [10]. In addition, ARDS management might also be complicated in pregnant women due to physiological pregnancy-related changes in the cardiorespiratory system such as reduced functional residual capacity and increased oxygen consumption [10]. Due to the patient’s severe obesity, she had a high risk of disease deterioration. Obesity is associated with altered pulmonary mechanics and physiology, with increased ACE2 expression, which is suspected to further increase the risk of respiratory failure in COVID-19 [11]. Various studies have shown that obesity and its associated complications, including hypertension and diabetes, are among the most crucial factors increasing the risk for a more serious course of COVID-19 requiring hospital admission and probably invasive ventilation [12, 13]. Other risk factors include older age, male sex, several ethnicities, smoking, COPD, malignant disease, immunodeficiency, and several coagulation disorders [14].

In our case, ECMO was considered an approach to improve the maternal respiratory condition and prolong pregnancy. The reported survival rates of pregnant women receiving extracorporeal life support are high. Although data reveal relatively low rates of severe complications in ECMO, complications such as major bleeding need to be considered [15, 16]. Prone positioning is known to be effective in improving oxygenation in the presence of ARDS; furthermore, it is suggested to be particularly beneficial in COVID-19 patients with moderate-to-severe disease [17]. In fact, pregnancy is not a contraindication for prone positioning when the pregnant anatomy and physiology are considered [17, 18].

Delivery should be considered in the management of refractory hypoxemic respiratory diseases [17]. In this case, fetal lung maturation was completed, while the estimated fetal weight promised adequate neonatal outcomes; thus, cesarean section was done to improve the maternal condition. A similar report of a pregnant woman with ARDS due to COVID-19 showed impressive improvement in maternal pulmonary function, with a significant increase in the P/F ratio shortly after delivery [19]. When treating our patient, we carefully assessed the maternal and fetal risks, and considered the lack of improvement in the patient’s oxygenation, in addition to considering the potential hypoxic damage to the fetus due to the ongoing maternal hypoxemia, potential complications of ECMO, and the benefits for the maternal cardiopulmonary condition as striking indications for delivery [20]. The patient’s respiratory state improved rapidly after delivery. Considering the fulminant course of COVID-19 in our patient, as well as the early gestational age, both outcomes were encouraging. Interestingly, we found that there was no vertical transmission of SARS-CoV-2, despite evidence of SARS-CoV-2-specific antibodies. The lack of transmission of IgG antibodies is most likely due to the antibodies being detected in the mother 9 days after delivery.

In conclusion, our case demonstrates that delivery should be considered in pregnant women with severe ARDS due to COVID-19 at an earlier gestational age and be provided in a tertiary setting, with an experienced multi-professional team. Since the management of ARDS is complex, it requires an individualized approach for decision-making that considers both maternal and neonatal outcomes.

## Data Availability

The datasets used and/or analyzed during the current study are available from the corresponding author on reasonable request.

## Abbreviations

ARDS: Acute respiratory distress syndrome
COVID-19: Coronavirus disease
ECMO: Extracorporeal membrane oxygenation
Ig: Immunoglobulin
SARS-CoV-2: Severe acute respiratory syndrome coronavirus 2
